# Characteristics of Patients Undergoing Laparoscopic Hysterectomy for Atypical Endometrial Hyperplasia

**DOI:** 10.7759/cureus.79856

**Published:** 2025-02-28

**Authors:** Shimpei Nagai, Shoko Kitazawa, Terumi Shirane, Asako Sera, Marie Fukutake, Tomomi Sakai, Yoko Fujioka, Makiko Hino, Yoshihisa Hattori, Takashi Kurahashi

**Affiliations:** 1 Obstetrics and Gynecology, National Hospital Organization Saitama Hospital, Saitama, JPN; 2 Obstetrics and Gynecology, Keio University, Tokyo, JPN

**Keywords:** atypical endometrial hyperplasia, endometrial cancer, laparoscopic surgery, mri, predicted factors

## Abstract

Objective: This study aimed to investigate the potential risk of a final pathological diagnosis of endometrial cancer in laparoscopic surgery for preoperatively diagnosed atypical endometrial hyperplasia (AEH), considering literature reports indicating a 40% coexistence rate of AEH with endometrial cancer.

Methodology: A retrospective analysis was conducted on 17 patients with preoperative AEH undergoing laparoscopic surgery at our hospital. The median age was 50 (37-74) years, and the median body mass index (BMI) was 25.1 (16.9-44.3) kg/m^2^. Surgical procedures included total hysterectomy and bilateral adnexectomy, accounting for the possible coexistence of endometrial cancer. Diagnostic methods comprised histological examination, dilatation and curettage, and pelvic MRI. Clinicopathological factors were thoroughly examined.

Results: Postoperative diagnoses were AEH in 10 cases, atypical polypoid adenomyoma (APAM) in one case, and endometrial cancer in six cases. Comparison between the AEH group and the endometrial cancer group showed that the proportion of postmenopausal women was higher in the endometrial cancer group (33.3% vs. 66.7%), as was the presence of endometrial thickening on imaging (20% vs. 66.7%), although these differences were not statistically significant. However, patients in the endometrial cancer group were significantly older than those in the AEH group (median age: 59.5 vs. 47.5 years, *P* = 0.02). All endometrial cancer cases were endometrioid carcinoma Grade 1, with five cases classified as FIGO (International Federation of Gynecology and Obstetrics) stage IA and one case as stage IB.

Conclusions: Despite nonsignificant differences in factors other than age, our study underscores the critical consideration of endometrial cancer during laparoscopic surgery for AEH, even with comprehensive preoperative examinations. This emphasizes the need for vigilant management strategies and heightened awareness of the surgical approach to AEH cases.

## Introduction

Atypical endometrial hyperplasia (AEH) is recognized as a precancerous lesion associated with endometrial cancer. Existing literature reports a prevalence of coexisting endometrial cancer in 17%-52% of patients undergoing hysterectomy for AEH [1-9]. Within our hospital, we have encountered instances where laparoscopic surgery was performed on patients preoperatively diagnosed with AEH, only to discover coexisting endometrial cancer in the final pathological diagnosis. While scattered reports discuss preoperative predictive factors for postoperative endometrial cancer diagnosis, a consensus remains elusive. This lack of unanimity complicates the formulation of a standardized diagnosis and treatment strategy. To address this gap, our study delves into the pathological factors and characteristics observed in cases diagnosed with endometrial cancer postoperatively following laparoscopic surgery for preoperative AEH. Through a focused examination of these factors, we aim to contribute valuable insights that may aid in refining diagnostic and therapeutic approaches in similar clinical scenarios.

## Materials and methods

Patients who were diagnosed with AEH by preoperative pathology and underwent laparoscopic total hysterectomy at the National Hospital Organization Saitama Hospital between May 2015 and June 2023 were included in this study. Patient background, chief complaint, pathological diagnosis, and advanced stage classification were analyzed by retrospectively extracting pathological factors and prognostic information based on outpatient, inpatient, and surgical medical records. After explaining it to each patient, we obtained written consent for the secondary use of medical information. In our hospital, when the preoperative diagnosis is AEH, we perform laparoscopic total hysterectomy and bilateral adnexectomy as the primary surgical procedures, taking into consideration the coexistence of endometrial cancer. In addition to histological diagnosis, curettage of the entire endometrium was performed in all cases possible, and pelvic contrast-enhanced magnetic resonance imaging (MRI) was performed as an imaging test. In cases where complete curettage could not be performed due to various factors, preoperative diagnosis was made only by outpatient histological examination, and rapid pathological diagnosis was added as appropriate according to intraoperative gross findings. In this study, patients with preoperatively diagnosed AEH who underwent total hysterectomy were classified into AEH and endometrial cancer groups based on the results of the final pathological diagnosis. A comparison was made regarding age at surgery, body mass index (BMI), CA125 level, endometrial thickening (20 mm or more before menopause/5 mm or more after menopause), and clinical background factors (menopausal status, etc.) in each group. The statistical analysis was performed using GraphPad Prism (Version 9, Dotmatics, Boston, MA) with the Mann-Whitney U test and Fisher's exact test, setting *P* < 0.05 as the significance level.

## Results

Seventeen patients with a preoperative diagnosis of AEH were included in the study. Patient background and chief complaints are shown in Table 1. The median age was 50 (37-74) years, the median BMI was 25.1 (16.9-44.3) kg/m^2^, and the median duration of observation was 37.3 (2.9-101.6) months (Table 1).

**Table 1:** Patient characteristics. BMI, body mass index

Characteristics (*n* = 17)		Values
Age (years) median (range)		50 (37-74)
BMI (kg/m^2^) median (range)		25.1 (16.9-44.3)
Observation period (months), median (range)		37.3 (2.9-101.6)
Pregnancy history	Yes	11
	No	6
Menopausal status	Before	10
	After	7

The most common chief complaint among patients was malignant genital bleeding (Table 2).

**Table 2:** Chief complaints.

Chief complaint (*n* = 17)	Values
Abnormal uterine bleeding	11
Abnormal cytology	2
Menstrual irregularities	2
Abdominal distension	1
Watery vaginal discharge	1

A comparison of postoperative pathological diagnoses is shown in Table 3.

**Table 3:** Comparison of postoperative pathological diagnoses. *P*-values were calculated between the AEH and endometrial cancer patient groups using the Mann-Whitney U test (*) and Fisher’s exact test (**). APAM (*n *= 1) was not included in the statistical analysis due to its unique pathological characteristics. AEH, atypical endometrial hyperplasia; APAM, atypical polypoid adenomyoma

	AEH (*n *= 10)	Endometrial cancer (*n *= 6)	APAM (*n *= 1)	*P*-value
Age (years), median (range)	47.5 (37-62)	59.5 (45-74)	43	0.03*
BMI (kg/m^2^), median (range)	26.1 (18.6-44.3)	25.4 (16.9-34.2)	19.7	0.64*
CA125 (U/mL), median (range)	12.1 (7.8-19.2)	15.3 (10.1-41)	13	0.48*
Nulliparous	5/10 (50%)	1/6 (16.7%)	No	0.31**
Menopause	3/10 (33.3%)	4/6 (66.7%)	No	0.30**
Thickened endometrium	2/10 (20%)	4/6 (66.7%)	No	0.12**

There were 10 patients in the postoperative AEH and six patients in the postoperative endometrial cancer group. The median age was 47.5 (37-62) years in the postoperative AEH group and 59.5 (45-74) years in the postoperative endometrial cancer group, and a significant difference was observed between the two groups. The median BMI was 26.1 (18.6-44.3) kg/m^2^ and 25.4 (16.9-34.2) kg/m^2^, respectively, showing no significant differences between the two groups. No significant differences were found in CA125, amenorrhea, post-menopause, and endometrial thickening between the two groups. There were six cases of postoperative diagnosis of endometrial cancer (Table 4).

**Table 4:** Characteristic of patients with endometrial carcinoma grade 1 (n = 6).

	Number of patients (*n *= 6)
Histological type	Endometrial carcinoma G1
Post-surgical stage	
IA	5
IB	1
Myometrial invasion	
No	4
<1/2	1
≧1/2	1
Vascular invasion	
Yes	1
No	5
Recurrence	0

The histological types were all endometrial carcinoma type G1, and the advanced stage of surgery (FIGO [10]) was stage IA in five cases and stage IB in one case. Four cases had no myometrial invasion, one case had less than 1/2 invasion, and one case had more than 1/2 invasion. No recurrence was observed in either the AEH group or the endometrial cancer group during this observation period.

## Discussion

In this study, 35% (6/17) of patients with a preoperative diagnosis of AEH were found to have coexisting endometrial cancer, similar to the results of previous reports [1-3]. There were no statistically significant differences in preoperative examinations or clinical background factors other than age among patients with a postoperative diagnosis of AEH or endometrial cancer. The rate of progression from AEH to endometrial cancer is said to be around 20% [11,12], and the coexistence rate of endometrial cancer in cases with a preoperative diagnosis of AEH is reported to be 27%-32.7% [13-16]. Endometrial histology is an important test for the diagnosis of endometrial cancer. Still, it may not be possible to obtain a specimen depending on the presence of organic diseases such as uterine fibroids or adenomyosis or the location of the lesion (uterine fundus or fallopian tube angle). As a means of supplementing endometrial histological diagnosis, close examination such as curettage and hysteroscopic biopsy may be performed. Still, it is reported that 32.7% and 45.3% of the cases could not be diagnosed by curettage and hysteroscopic biopsy, respectively [13]. In the six cases that were diagnosed as endometrial carcinoma by the final pathological diagnosis, five cases showed lesions at the uterine fundus, and one case showed a lesion at the left fallopian tube angle. Despite preoperative complete curettage in all cases, cancer could not be diagnosed. In the case of a lesion at the angle of the fallopian tube, a hysteroscopic biopsy was performed at the same time, but it did not lead to the diagnosis of cancer (Table 5).

**Table 5:** Patient characteristics and location of lesions in endometrial cancer cases in this study.

Case	Age (years)	Menopausal status	Pregnancy history	BMI	Symptoms	CA125 (U/mL)	Endometrial thickness (mm)	Primary location
1	60	Pre-menopausal	G2P2	27.2	Abnormal uterine bleeding	15.1	22	Polypoid lesion in the fundus
2	52	Post-menopausal	G3P3	27.4	Abnormal pap smear	10.1	21	Raised area in the fundus
3	74	Post-menopausal	G2P2	16.9	Abdominal mass	15.5	5.9	A lesion with an 8 mm lump in the fundus
4	73	Post-menopausal	G4P2	22.2	Abnormal uterine bleeding	10.6	20	An unclear lesion, slight abnormality in the fundus
5	45	Pre-menopausal	G0P0	23.6	Watery discharge	18.1	25	A lesion with a 30 mm lump in the fundus
6	59	Post-menopausal	G2P2	34.2	Abnormal uterine bleeding	41	6	Slight lesion in the posterior wall left fallopian tube angle

Based on these results, it is necessary to recognize that diagnosis is difficult depending on the location of endometrial lesions and consider the possibility of endometrial cancer when operating on a patient with a preoperative diagnosis of AEH. In addition, it has been reported that CA125 ≥ 35 U/mL, post-menopause, and endometrial thickening (≥20 mm) are independent prognostic factors for postoperative diagnosis of endometrial cancer in patients with a preoperative diagnosis of AEH [9,17]. In the present study, none of these factors showed significant differences. Still, the postoperative endometrial cancer group tended to have higher scores than the postoperative AEH group in the postmenopausal and endometrial thickening categories (Table 1). At our hospital, we evaluate the presence or absence of tumors and myometrial invasion by contrast-enhanced MRI of the pelvic region before surgery on the assumption that the patient may have endometrial cancer. According to previous reports, the correct diagnosis rate of myometrial invasion of endometrial cancer by pelvic contrast-enhanced MRI is about 80% [18]. However, there are reports that myometrial invasion is underestimated in cases with uterine myoma or uterine adenomyosis [19]. In this study, the myometrial invasion was unclear in a patient with stage IB disease because the patient also had adenomyosis. In this study, the myometrial invasion was considered unclear in stage IB because the patient also had adenomyosis. From the above, we believe that even if these imaging examinations, including transvaginal ultrasonography, are performed before surgery, the possibility of underestimation, including myometrial invasion, should be considered, and surgery should always be performed assuming the coexistence of endometrial cancer. In terms of surgical operation, after collecting cytological cytology at the beginning of the surgery, bilateral fallopian tubes are sealed, and a uterine manipulator is inserted as appropriate. The paraspinal connective tissue is processed outside the myometrium, and if vaginal retrieval is complex due to uterine fibroids, etc., it is placed in a retrieval bag, and the incision at the umbilical region is extended and retrieved without making a fine incision. This study was retrospective, and the number of cases was not significant, so the estimation of prognostic factors was limited. In addition, the pathological evaluation of AEH and endometrial carcinoma grade 1 is complex, and the judgment may differ depending on the institution and the pathologist's experience. We believe it is necessary to improve the quality of diagnosis and research by accumulating more cases and conducting a joint evaluation with radiologists and pathologists for more detailed studies.

## Conclusions

Among the patients diagnosed with AEH preoperatively, 35% were diagnosed with endometrial cancer postoperatively, and in one case, stage IB cancer was also diagnosed. Although there was no significant difference in prognostic factors other than age in this study, the results reaffirm the need to consider the possibility of a postoperative diagnosis of endometrial cancer even in patients diagnosed with AEH after thorough preoperative examinations.
